# Estimation of mediators in the associations between campus green spaces and students’ anxiety: a case study in Nanjing

**DOI:** 10.3389/fpsyg.2024.1396548

**Published:** 2024-10-10

**Authors:** Wanting Diao, Silei Li, Bing Zhao, Fan Zhang

**Affiliations:** The College of Landscape Architecture, Nanjing Forestry University, Nanjing, China

**Keywords:** campus green space, NDVI, anxiety, mental health, pathways

## Abstract

**Introduction:**

Although a number of scholars have examined the theoretical pathways between green space (GS) and mental health, few have focused on how campus greenness affects the mental health of Chinese youth.

**Methods:**

Herein, two objective indicators, campus and individual 300-m normalized vegetation index (NDVI) data, were used as independent variables. A questionnaire was used to collect the self-rated anxiety level of students on campuses in Nanjing. Then, we chose “subjective perception of campus GS”, “physical activity”, “social cohesion”, “nature relatedness” and “usage pattern” as mediating variables to explore the pathways between the campus greenery and college student’ anxiety level through correlation analysis, linear regression, and mediation effect test.

**Results:**

Results showed the campus-wide NDVI and individual students’ 300-m range NDVI had significant negative correlations with anxiety (*p* = 0.045, *p* = 0.023). Campus perception, nature relatedness and the frequency of using GS are the pathways through which campus GSs influence student anxiety.

**Discussion:**

Our findings emphasised the importance of subjective perceptions of greenspaces, which provided a direction that can be deepened in future research.

## 1 Introduction

Urban green spaces (GSs) are the basic elements of human settlements, including urban parks, street green spaces, community gardens, and rooftop and vertical greening. They play an important role in natural ecosystems, providing ecological functions such as air purification, climate regulation, heat island effect mitigation, and biodiversity preservation (Mukherjee and Takara, 2018; Yin et al., 2022). Therefore, urban green spaces are an important factor for the sustainable development of cities (Mukherjee and Takara, 2018; Kuklina et al., 2021). In recent decades, numerous studies have demonstrated that natural urban environments have potential health benefits, and a link has been established between the environment and health (Triguero-Mas et al., 2015; van den Berg et al., 2015; Bratman et al., 2019). Exposure to nature can reduce stress and mental illness, provide health benefits, and increase life satisfaction (Hartig et al., 2014; Triguero-Mas et al., 2015; Bratman et al., 2019; Zhang et al., 2020; Cheng et al., 2021; Nguyen et al., 2021). A study on youth and student populations reported that the level of greening is positively correlated with the level of GS perception. Improving the quality of greenery can reduce people’s anxiety and increase stress resistance (Dzhambov et al., 2019). Therefore, exposure to GS is recognized as a nature-based solution to improving people’s mental health while promoting ecological sustainability (Wolch et al., 2014; van den Bosch and Sang, 2017; Yu et al., 2020).

Based on many previous studies, we can categorize the pathways of the effects of urban green space on health into five aspects, which are green space perception, green space use pattern, social cohesion, physical activity, and nature relatedness (Stigsdotter et al., 2010; Nisbet and Zelenski, 2013; Zhang et al., 2017; Dzhambov A. M. et al., 2018; Ma et al., 2018; Jennings and Bamkole, 2019; Grilli et al., 2020). The selection of the five mediating pathways above was grounded in a comprehensive theoretical framework derived from existing literature on environmental psychology and public health.

Green space perception can be understood as people’s views and feelings about the use of green space, such as whether they are satisfied with the area, layout, esthetics, and plant richness of green space. It may serve as a link between greenness and anxiety. Perceived GS quality can not only promote life satisfaction and wellbeing but also mediate GS and health (Zhang et al., 2017). One study showed that a community’s perceived greenness is positively correlated with physical and mental health scores (Akpinar, 2016). One study focussing on college students identified four dimensions: green comfort, reasonable layout, beautiful scenery, and diverse plants, as criteria for assessing college students’ subjective recognition of campus GS. The study found that campus GSs had a positive impact on mental health, with a greater effect on the male student population than on the female population (Liu W. et al., 2022). Research studies on environmental satisfaction in residential areas showed that people’s overall satisfaction with community GS in both developed and developing countries has a strong relationship with mental health (Dong and Qin, 2017; Ma et al., 2018; Liu et al., 2019).

The second pathway linking greenness to anxiety is the patterns of GS use, which include frequency of use of green space, duration of each use on average, average frequency, and duration of seeing green views through windows per day. The frequency and duration of each use of GS are proven to be related to self-reported health (Nielsen and Hansen, 2007; Stigsdotter et al., 2010; Grilli et al., 2020). A study of college students showed that frequency of use has a more favorable impact on health (Liu Q. et al., 2022), and a significant relationship is found between travel patterns and mental health issues (Bai et al., 2024). Moreover, individuals who use GSs for longer periods of time tend to be in better moods and have lower levels of perceived stress (Holt et al., 2019). Close contact with the natural environment is particularly important for improving poor psychological states (van den Berg et al., 2017; Hubbard et al., 2021), corroborating Hartig’s view that natural contact is a prerequisite for the pathways (Hartig et al., 2014). Furthermore, the study found that students’ use of and participation in campus GS facilities, as well as participation in campus activities, can enhance their emotional and cognitive domains (McFarland et al., 2010). Studies show that indirect exposure of residents to GSs through windows in their homes was assessed for an association with health and suggested that having a green window view can have a mood-moderating effect (van Dillen et al., 2012; Bi et al., 2022). Evidence indicated that indirect exposure to GS—having a green window view—can have a mood-moderating effect (Li and Sullivan, 2016).

The third pathway that links greenness to anxiety is related to social cohesion. Social cohesion can affect health through social participation, support, influence, and interaction (connectedness) (Jennings and Bamkole, 2019). Studies found that GSs that provide restorative environments may attract residents to spend time outside for social interactions. This can enhance residents’ sense of community belonging (Dzhambov A. et al., 2018). Dadvand et al. also observed that social support mediates the effect between the quality of community GS and residents’ wellbeing (Dadvand et al., 2016). In Foellmer et al.’s (2021) Healthy Academic Greenspace Framework, good social relationships and physical activity were found to be effective in enhancing students’ wellness and facilitating the integration of positive life experiences into their daily learning and working environments (2020).

The fourth pathway linking greenness to anxiety relates to physical activity. In research on mental health in youth populations, physical activity and social cohesion were found to mediate the link between GSs and mental health (Dzhambov A. et al., 2018). Green space is an ideal place to engage in physical activity for the public because it is safe, easy to reach, and attractive (Mytton et al., 2012). Evidence shows that GSs are better places for physical activity to improve mental and physical health (Mitchell, 2013). Moreover, living near urban GSs encourages physical activity, such as walking, which provides significant psychological and physiological benefits to the public (Sugiyama et al., 2008; Cohen-Cline et al., 2015). A study of Chinese college students suggests that physical activity can alleviate the anxiety associated with examinations (Tian et al., 2024). The frequency of physical activity is negatively associated with a person’s mental health outcomes; the duration of physical activity is positively associated with a person’s physical health outcomes. In addition, large open/visible urban GSs are related to better health results (Akpinar, 2016). Holt et al. (2019) examined active and passive GS use behaviors and found that students who regularly engage in GS activities (e.g., participating in sports and socializing with friends) were less likely to feel stressed and showed better emotional states.

The fifth pathway linking greenness to anxiety is nature relatedness. Nature relatedness is a psychological characteristic reflecting people’s connection to nature. It contains several dimensions such as cognitive processes, personal experience, and behavior (Dean et al., 2018). This concept is playing an increasingly important role in studies of environmental behavior, mental health, and wellbeing (Nisbet and Zelenski, 2013). Existing campus research studies focussed more on student perceptions of natural environments. Liu Q. et al. (2022) observed significant associations between college student perceptions of the naturalness of the campus, as well as the frequency and duration of use of campus GSs, with perceptions of naturalness also having a positive impact on mental and physical health. The research investigated the relationship between students’ perceptions of species richness in the natural environment of the campus and their emotional states. Participants watched a video of the campus environment, and the findings indicated that high species richness reduced negative emotional states (Ha and Kim, 2021). The intrinsic relationships among student health, mood, and nature in campus environments require further exploration.

Mental health problems have been recognized as being globally prevalent among contemporary youth populations. The 2022 National Education Statistics showed that over 20% of Chinese college students suffer from varying degrees of depression, with 18.5% exhibiting tendencies toward depression, 4.2% at high risk of depression, and 8.4% showing signs of anxiety. Notably, these rates have progressively increased over the past decade (Gao et al., 2020; Fu et al., 2021). An official report from China (Fu et al., 2021) shows that young people aged 18–34 are more likely to be anxious than other adults, while the mental health index of the 18–25 group is lower than that of all other age groups. Figures for 2021 to 2022 (Fu et al., 2023) also show that young adults, especially those aged 18–24, are at high risk of depression, with a detection rate of 24.1%. Thus, mental health problems that are more prevalent in youth need to be prevented and intervened.

With the development of campus greenery, preliminary research has investigated the health benefits that campus GSs bring to the quality of life, physical fitness, and restorative perceptions of students (Hipp et al., 2016; Liu W. et al., 2022). However, there is insufficient research on its deeper mechanisms of action. Further research on the action pathways is important for the future guidance of campus green space planning and construction. Furthermore, although several studies in China demonstrated a positive correlation between greenery around cities and mental health, such as in Beijing and Guangzhou (Zhang et al., 2018; Xu et al., 2021), there is a paucity of empirical research on the relationship between campus GSs and mental wellbeing in China, and it remains unclear how campus GSs affect mental wellbeing. Therefore, we want to explore whether the five pathways (campus perception, physical activity, social cohesion, nature relatedness, and GS usage patterns) summarized above can play a mediating role in the relationship between campus green exposure and students’ anxiety level, and we designed a conceptual modeling framework (Appendix Figure 1). To investigate these potential pathways, we used survey data from 18 campuses in the Nanjing area to explore the pathways of action between campus GS exposure and anxiety among Chinese college students. We investigated whether these associations (if any) are moderated by campus size, number of students enrolled, number of bus and subway point of information (POI), number of dining and entertainment life service POIs within 1,000 m of each entrance/exit, and moderated by gender, age, grade, major, and average monthly cost of living. In this article, we seek to explore the following research questions:

1. Is there a correlation between campus green space and anxiety among college students?
2. What are the pathways in which campus green spaces influence college students’ anxiety?
3. Do students’ behavioral patterns of using green spaces play a role?

## 2 Methods

### 2.1 Data resource

A questionnaire survey (Appendix Table 1) was conducted between October and November 2022 in Nanjing, China. Nanjing is an education centre in eastern China with the number of universities being second only to Beijing and Shanghai. We randomly selected 20 campuses from the 43 campuses of 33 universities in six districts of Nanjing as the study sample (Appendix Figure 2). Electronic questionnaires were distributed on campus by scanning the QR codes of the questionnaires. Current students over the age of 18 were eligible to participate in the study, while minors, outsiders, or other employees of the campus were not eligible to complete the questionnaire. Before administering the questionnaire, we checked whether the person was a student at the school and whether they were above 18 years of age. A total of 821 questionnaires were obtained. The questionnaire consisted of four main sections (see Appendix Table 1): (1) information on respondent sociodemographic characteristics (gender, age, grade level, area of specialization, and average monthly cost of living); (2) patterns of campus GS use (frequency and duration of spontaneous and direct use of campus GS, perceived accessibility, and frequency and duration of indirect use of GS); (3) perceived status of the respondents, which included the subjective perception of campus GS, physical activity, social cohesion, and nature relatedness; and (4) anxiety data of respondents, using the 7-item Generalised Anxiety Disorder Scale. A total of 654 valid questionnaires were obtained from the 18 campuses, exceeding the minimum sample size of 472 calculated using G-Power. The questionnaires that took less than 1 min to complete and those with missing information were excluded as were questionnaires from school districts with fewer than 15 questionnaires. In addition, in the future, we plan to collect longitudinal data to conduct follow-up studies to examine changes between GS and student anxiety on campus.

#### 2.1.1 Mental wellbeing outcomes

The questionnaire used the Generalised Anxiety Scale (GAD-7) to obtain data on student self-assessed anxiety. The GAD-7 is a brief, validated self-assessment of anxiety consisting of seven items (e.g., “difficulty relaxing” and “inability to stop or control worrying”); each item has four options representing different frequencies of anxiety symptoms. Each question is scored on a scale from 0 to 3. The scores of the seven items were added together to finally reflect the anxiety level of the subjects in the form of an overall score ranging from 0 to 21(minimal 0–4, mild 5–9, moderate 10–14, and severe 15–21) (Mills et al., 2014; Seo et al., 2014). The scale was proved to have good reliability and validity and is widely used in clinical practice and research studies to rapidly assess generalised anxiety disorder (Spitzer et al., 2006). Cronbach’s coefficients indicated high internal consistency (>0.80) among the seven items.

#### 2.1.2 Campus GSs and 300-m range individual normalised vegetation index data

In this study, the normalized vegetation index (NDVI) was chosen as an objective indicator of campus GS exposure. NDVI is a remote-sensing analytical tool to quantify the green density by measuring the difference between the near-infrared radiation reflected and red light absorbed by healthy green vegetation. Gulwadi et al. measured the amount of objective greenness of a campus through the NDVI at three spatial levels: the campus as a whole, the central area of the campus, and the periphery of the academic buildings, and analysed the association between the objective greenness at each level and the students’ perception of the campus green environment, perceived restorativeness, and living standard (Gulwadi et al., 2019). This suggests that subjective perceptions and objective measurements can reveal different aspects of green space information. Therefore, we measured the campus NDVI and individual 300-m individual NDVI to analyse the association between the environment and emotion more accurately.

Campus NDVI data were obtained from Sentinel-2A remote-sensing satellite images with a resolution of 10 m. Furthermore, the remote-sensing images of Nanjing in the fall during the period from 2018 to 2021 were selected as the main data source. After preprocessing the raw data, Arc GIS 10.3 was used to plot the campus boundary and extract the average NDVI value of the campus area. The questionnaire survey obtained the number of dormitory buildings or academic buildings where the individual students in the surveyed sample spent most of their time daily. We used Baidu’s map coordinate picking system^1^ to get the latitude and longitude of the geographical coordinates of the individual samples based on the location of the building number. The converted coordinate points were imported into Arc GIS 10.3 via QGIS, and a 300-m buffer range for individual students was obtained using the domain analysis tool. NDVI data were then superimposed on the buffer range to extract the average NDVI data of the 300-m range of each individual.

### 2.2 Variables

#### 2.2.1 Mediating variables

We assessed five mediators between campus GSs and student anxiety. The first medium is the respondents’ perception of the campus green space. The evaluation consists of four parts: comfortable greenery, a rational layout, beautiful scenery, and abundant plants (Liu W. et al., 2022), where Cronbach’s alpha was 0.761. Second, physical activity was determined using the International Physical Activity Scale short version (IPAQ-short). This scale gaged respondents’ daily physical activity levels in terms of strenuous, moderate, and walking exercises, as well as sedentary time duration (Hagstromer et al., 2006; Papathanasiou et al., 2010). The reliability of the scale, measured using Cronbach’s alpha, was 0.630. Third, the Social Cohesion Scale was used to evaluate social relationships and cohesion from four perspectives: trust, attachment, tolerance, and respect (Dzhambov A. M. et al., 2018). Fourth, the nature relatedness scale was used to measure the affective and experiential aspects of an individual’s connection to nature (Nisbet and Zelenski, 2013; Robinson et al., 2021; Liu Y. et al., 2022); Cronbach’s alpha was 0.815. Finally, the green space use pattern captured the use and perception of GSs of the respondents from three perspectives: frequency and duration of spontaneous direct use of campus GSs, frequency and duration of indirect use of GSs, and perceived accessibility (Liu Q. et al., 2022).

#### 2.2.2 Control variables

We collated a range of covariates, including information on respondents’ sociodemographic characteristics and several objective control variables. Summary statistics for all variables are presented in Table 1.

**Table 1:** Summary statistics.

Variables	Description	Proportion/Mean (SD)
Key variables		
GAD-7	Respondents’ self-reported level of anxiety (score range 1–21)	7.090 (4.696)
“No anxiety”	0–4	35.5
“Mild anxiety”	5–9	26.3
“Moderate anxiety”	10–14	34.6
“Severe anxiety”	15–21	3.7
Campus green space NDVI	Continuous variable (−1–1)	0.267 (0.033)
300-m range individual NDVI	Continuous variable (−1–1)	0.255 (0.041)
Campus green space perception (CGS)		4.142 (0.642)
Physical activity (PA)		2.960 (0.691)
Social cohesion (SC)		5.865 (0.892)
Nature relatedness (NR)		4.095 (0.617)
Usage pattern (UP)		
Duration of viewing GS (through windows)		2.927 (0.928)
Duration of using GS		3.139 (0.808)
The time it takes to get to GS		2.835 (0.906)
Frequency of viewing GS (through windows)		2.847 (0.872)
Frequency of using GS		2.994(0.892)
Socioeconomic covariates		
Age (18–20)		82.3
Age (>20)		17.7
Gender (men)		52.3
Gender (women)		47.7
Grade (freshman)		10.4
Grade (sophomore)		40.8
Grade (junior)		29.8
Grade (senior)		9.9
Postgraduate or above		9.0
Professional relevance	Relevance of major studied to green space	
Completely irrelevant		7.3
Relatively irrelevant		9.9
Median		14.8
Relatively relevant		36.9
Completely relevant		31.0
Monthly expenses (<1,000 CNY)		3.1
Monthly expenses (1000–2000 CNY)		34.7
Monthly expenses (2000–3,000 CNY)		52.0
Monthly expenses (3000–5,000 CNY)		8.9
Monthly expenses (≥5,000 CNY)		1.4
Other covariates		
Campus area		116.104 (81.799)
Number of students		20008.970 (9437.979)
POI number of traffic	Number of bus and subway POIs within 1,000 m of school entrances and exits	11.540 (6.777)
POI number of catering and entertainment	Number of POIs for dining and entertainment lifestyle services within 1,000 m of school entrances and exits	373.000 (297.056)

### 2.3 Statistical analyses

Pearson’s correlation index *r* ranges from −1 to +1, where 0 indicates no linear relationship between two variables, −1 indicates a perfect negative correlation, and + 1 indicates a perfect positive correlation (Adler and Parmryd, 2010).

Multiple linear regression analyses using SPSS 26.0 were used to test whether the variables significantly predicted anxiety in college students. We tested the direct effects of campus GSs and individual 300-m NDVI on anxiety (Table 2).

**Table 2:** Multiple linear regression analyses.

Model 1: Campus NDVI								
Dependent variable	Independent variable	*B*	SE	Beta	*t*	*p*	VIF	*R* ^2^
Anxiety	(constant)	**11.258**	**1.791**		**6.287**	**0.000**		0.047
	Campus NDVI	**−11.105**	**5.525**	**−0.078**	**−2.010**	**0.045**	1.019	
	Area	**−0.012**	**0.003**	**−0.201**	**−3.991**	**0.000**	1.717	
	POI number	**−0.003**	**0.001**	**−0.197**	**−3.813**	**0.000**	1.820	
	Age	0.140	0.513	0.011	0.272	0.786	1.187	
	Monthly expenses	0.418	0.251	0.065	1.666	0.096	1.024	

Model 2: 300-m NDVI								
Dependent variable	Independent variable	*B*	SE	Beta	*t*	*p*	VIF	*R* ^2^
Anxiety	(constant)	**11.065**	**1.585**		**6.980**	**0.000**		0.049
	300-m NDVI	**−10.556**	**4.648**	**−0.092**	**−2.271**	**0.023**	1.111	
	Area	**−0.011**	**0.003**	**−0.187**	**−3.686**	**0.000**	1.760	
	POI number	**−0.003**	**0.001**	**−0.210**	**−4.053**	**0.000**	1.836	
	Age	0.096	0.512	0.008	0.188	0.851	1.183	
	Monthly expenses	0.402	0.251	0.062	1.601	0.110	1.028	

Bold values represent results that are significant.

Mediated effect analyses were then used to assess five theoretically indicated pathways linking different dimensions of various indicators related to campus GSs to anxiety symptoms based on previous approaches (Igartua and Hayes, 2021), and the indirect effects in the mediation model were calculated. The hypothesized mediating effects between campus-wide NDVI and student anxiety were tested using Mplus 8.3, employing maximum likelihood estimation and 5,000 bootstrap samples.

## 3 Results

### 3.1 Descriptive analyses and correlations among the variables

Participants were 52.3% men and 47.7% women. The majority of participants (82.3%) were between 18 and 20 years old, and the number of undergraduate college sophomores (40.8%) and juniors (29.8%) was relatively large. Monthly spending was categorized into five groups: 3.1% of participants spent <1,000 CNY per month; 34.7% spent between 1,000 and 2,000 CNY per month; 52.0% spent between 2,000 and 3,000 per month; and a small percentage (8.9 and 1.4% respectively) spent between 3,000 and 5,000 CNY per month and > 5,000 CNY per month. More than half (67.9%) majored in GSs. For anxiety, 35.5% reported “no anxiety” (i.e., GAD-7 score ≤ 4), and 3.7% reported “severe anxiety” (i.e., GAD-7 score ≥ 15).

Pearson’s correlations between the variables are shown in Appendix Figure 2. Red represents a negative correlation, and blue represents a positive correlation, with darker colors indicating a stronger correlation. Campus-wide NDVI and individual student 300-m-wide NDVI were negatively correlated with anxiety, indicating that greater vegetation cover and a greener environment are associated with lower levels of anxiety among students. The NDVI at the 300-m range of an individual student had a slightly greater effect on student anxiety than the NDVI in the campus-wide range. In the correlation analysis of mediator variables, campus perception, physical activity, social cohesion, and nature relatedness were negatively correlated with anxiety, with coefficients of −0.317, −0.149, −0.300, and −0.268, respectively.

Among the campus GS use patterns, “frequency of use” and “duration of viewing” were negatively correlated with anxiety, and the correlation coefficients were −0.145 and −0.13. Perception of campus GSs was positively correlated with social cohesion and nature relatedness, and social cohesion had significant positive correlations with nature relatedness; campus-wide NDVI had significant positive correlations with 300-m-wide NDVI. Campus area had a significant negative correlation with the number of traffic POIs and other service facility POIs, while the three variables, namely, “duration of use of GS,” “duration of perceived accessibility of GS,” and “frequency of viewing window views,” were not significantly correlated with anxiety and were therefore not included in the subsequent analysis. Among the control variables, campus area, number of POIs such as dining and entertainment, age, and average monthly cost of living showed significant correlations with anxiety, whereas the other variables did not show correlations and were excluded from subsequent analyses.

### 3.2 Multiple linear regression analyses

Table 3 presents the relationship between campus GSs and anxiety levels. According to the results of the regression analysis, campus-wide NDVI and individual 300-m-wide NDVI were significantly negatively correlated with student anxiety. According to the standardized regression coefficients and R2 values, the individual student 300-m-wide NDVI had a greater effect on anxiety than campus-wide NDVI. For model 1, the R2 was 0.047 with the addition of control variables, and the absolute value of t for campus NDVI, campus area, and number of POIs were all >1.96, with a *p*-value of <0.05. In addition, none of the 95.0% confidence intervals for B contained 0, indicating that campus NDVI, campus area, and number of POIs affected student anxiety in this model, with unstandardised coefficients of −11.105, − 0.012, and − 0.003, respectively. For model 2, the R2 was 0.049 with the addition of control variables, the absolute value of t was >1.96 for 300-m-wide NDVI, campus area, and number of POIs, with a p-value of <0.05, and none of the 95.0% confidence intervals of B contained 0, indicating that the 300-m range NDVI, campus area, and number of POIs had an effect on student anxiety in this model with unstandardised coefficients of −10.556, −0.011, and − 0.003, respectively.

**Table 3:** Serial mediation effect tests between NDVI and anxiety symptoms.

Path relationship	Point estimates	SE	*p*-value	Bootstrap 5,000 times 95% CI			
				Bias corrected		Percentile	
**CN→CGS→ANX**	**−0.984**	**0.325**	**0.002**	**−1.722**	**−0.427**	**−1.683**	**−0.394**
CN → PA → ANX	−0.195	0.146	0.182	−0.577	0.013	−0.540	0.031
CN → SC → ANX	−0.677	0.270	0.441	0.012	−1.316	−0.211	−1.259
**CN→NR→ANX**	**−0.591**	**0.248**	**0.017**	**−1.211**	**−0.193**	**−1.150**	**−0.163**
**CN→FRE→ANX**	**−0.231**	**0.109**	**0.034**	**−0.497**	**−0.066**	**−0.472**	**−0.053**
CN → DUR → ANX	−0.205	0.106	0.053	−0.491	−0.050	−0.446	−0.035
**IN→CGS→ANX**	**−0.632**	**0.240**	**0.008**	**−1.176**	**−0.215**	**−1.145**	**−0.204**
IN→PA → ANX	−0.081	0.076	0.284	−0.324	0.006	−0.266	0.023
IN→SC → ANX	−0.313	0.197	0.112	−0.738	0.039	−0.731	0.043
**IN→NR→ANX**	**−0.386**	**0.176**	**0.028**	**−0.794**	**−0.089**	**−0.774**	**−0.075**
IN→FRE → ANX	−0.034	0.075	0.647	−0.210	0.098	−0.195	0.112
IN→DUR → ANX	−0.148	0.084	0.079	−0.336	−0.027	−0.342	−0.016

CN, campus NDVI; CGS, campus green space perception; PA, physical activity; SC, social cohesion; NR, nature relatedness; FRE, frequency of using CN, campus NDVI; CGS, campus green space perception; PA, physical activity; SC, social cohesion; NR, nature relatedness; FRE, frequency of using campus green spaces; DUR, duration of window view; IN, individual 300-m-wide NDVI; ANX, anxiety symptoms.

### 3.3 Serial mediation effect analyses

The results of serial mediation effect tests between NDVI and anxiety symptoms are shown in Table 3. We found five significant paths: campus NDVI → campus GS perception → students’ anxiety (PE = −0.984, *p* = 0.002); campus NDVI → nature relatedness → students’ anxiety (PE = −0.591, *p* = 0.017); campus NDVI → frequency of using GS → students’ anxiety (PE = −0.231, *p* = 0.034); 300-m range NDVI → campus GS perception → students’ anxiety (PE = −0.632, *p* = 0.008); and 300-m range NDVI → nature relatedness → students’ anxiety (PE = −0.386, *p* = 0.028), while “frequency of using GS” was not significant in the path 300-m NDVI → frequency of using GS → students’ anxiety (PE = −0.034, *p* = 0.647). The indirect effects of physical activity, social cohesion, and duration of using GS were not significant (*p* > 0.05), indicating that mediation was not established between campus NDVI and individual NDVI in relation to anxiety. Additionally, the 300-m NDVI’s effect on the frequency of use concerning anxiety was also not significant, further confirming the lack of mediation.

## 4 Discussion

Taking Nanjing universities as an example, this study combines empirical research to explore the effects of campus GSs on college students’ anxiety. We analysed the pathways in which campus GSs affect college students’ anxiety, finding a significant negative correlation between NDVI and student anxiety symptoms both at the campus scale and 300 m of the individual, thereby addressing the research question 1. According to mediation effect analysis, the campus-wide NDVI can influence student anxiety through campus perception, social cohesion, nature relatedness, and frequency of viewing in green space use patterns. The individual 300-m range NDVI can influence student anxiety through campus perception and nature relatedness, which provides a definitive answer to research questions 2 and 3.

Based on Pearson’s correlation analysis and linear regression results, the correlation size and R^2^ values are not very high. This suggests that although there is a link between campus green space and students’ anxiety, green space will not be the main or important factor affecting students’ anxiety. On the one hand, present-day young people are not very interested in green spaces, and in an era of advanced networks and abundant recreational activities, few would choose to actively seek out green spaces in their free time; one study (Wang et al., 2023) also pointed out that the lack of interest in green space activities among young people may be caused by unsatisfactory environmental cleanliness and spatial quality of the activity space. On the other hand, there are individual differences in how campus green spaces impact students’ mental health. Factors such as students’ habits, upbringing, interests, and academic workload may influence this relationship.

According to our results, GS exposure was significantly related to respondent self-rated mental health outcomes, which is consistent with previous studies (Zhang et al., 2017; Jennings and Bamkole, 2019; Wang et al., 2019; Liu Q. et al., 2022; Zhang et al., 2023). We also confirmed the existence of three pathways between GS exposure and mental health: green space perceptions, nature relatedness, and the frequency of using GSs. These have supported evidence from a number of previous studies (Martyn and Brymer, 2016; Ha and Kim, 2021; White et al., 2021; Liu W. et al., 2022; Yang et al., 2023). Through the questionnaire and data analysis section, we can easily see that college students are eager to establish a connection with nature, and many of them strongly agree that “our relationship with nature is an important part of who we are.” Thus, we support the idea that “relating to nature may be a pathway for human wellbeing and environmental sustainability” (Zelenski and Nisbet, 2014). This also explains, to some extent, why the effect of campus subjective perception on students’ anxiety is also more significant. Because college students care about the relationship between individuals and nature, they would like the campus green space to be spacious and comfortable, rationally arranged, beautiful, and rich in plants, and the better the campus green environment is in these aspects, the more it can alleviate students’ anxiety. Our findings supported the theory that natural environments, such as campus GSs, play a crucial role in psychological restoration and stress reduction and added empirical evidence to the biophilia hypothesis, which suggests that humans have an innate affinity for nature. In addition, our findings highlight that personal 300-m-wide NDVI is more important for student anxiety than campus-wide NDVI, which requires that landscape planners should not only focus on the design of green spaces at large scales around the campus but also consider the detailed environments that students encounter in their daily lives. The level of importance students show for natural connections also suggests that university campus designers should add more interactive green space amenities. However, our results are not in accord with previous studies (Dzhambov A. et al., 2018; Jennings and Bamkole, 2019; Grilli et al., 2020) indicating that GSs cannot provide psychological health benefits by improving social cohesion, physical activities, and social cohesion. We suspect that more confounding factors may need to be taken into account. For instance, a study has pointed out that individuals’ mental health is influenced by whether they have a partner (Bai et al., 2024). A review (Aghabozorgi et al., 2024) has noted that the impact of the university landscape on mental health may depend on different types and characteristics of spaces. Therefore, distinguishing between different types of green spaces in such studies may be necessary and beneficial to better advise health-oriented campus environment design. We plan to include this distinction in our future research.

Our study extends previous research on the relationship between greenery and mental health in several ways. First, based on existing research on the health benefits of GSs (2020), we further explored the mechanism of campus GSs on college student anxiety. The relationship between urban GSs and health is relatively mature; however, empirical studies on campus GSs are insufficient (Hartig et al., 2014; Markevych et al., 2017; Dzhambov A. et al., 2018); this study helps to fill this gap and confirms the health benefits of campus GS. Second, the influence mechanism of campus GSs and college student anxiety was explored at two levels: campus-wide and within a 300-m range of individual students. This approach involved analyzing GS indicators for the overall campus as well as detailed indicators within the areas of daily activities of individual students. College campuses are often large, and indicators of students’ daily activity range can more accurately analyse the association between the environment and emotion. Third, we have taken into account subjective factors in addition to objective ones. The previous study categorized the mediators of GSs affecting residents’ mental health as “environmental factors,” “outdoor activities,” and “social cohesion” (Chen et al., 2021); on this basis, we used “GS perceptions” and “GS use patterns” as mediators instead of “environmental factors.” To determine which factor plays a more important role—subjective (green space perception) or objective—we added a regression analysis that included both factors (Appendix Table 2). The results indicated that green space perception showed greater significance when compared with monthly expenses, campus area, POI number of catering and entertainment, age, NDVI, and other variables. This proved that subjectively perceived factors are more important than objective ones. In addition, we added “nature relatedness” as a new pathway between GS exposure and anxiety. Combining student subjective perceptions of campus GSs for greening, layout, and esthetics in the research process, and incorporating subjective and objective factors into the analysis, we found that the objective indicators of campus GSs influenced other mediating factors by acting on subjective perceptions, which consequently influenced students’ anxiety level.

There are some research limitations in this study. First, we used GS indicators that were data collected through remote-sensing information, which still has a gap with the real campus GSs that the student population is exposed to, and it is difficult to avoid this error. More refined indicators should be considered in future studies to approximate the real spatial status. Second, the different study areas may have certain impacts, and in different urban environments and geographical contexts, the health benefits generated by GSs may differ. The study area was located in Nanjing, and the results are not representative of all regions. The inclusion of research sites in northwest China, Northeast China, and the Central Plains may increase the comprehensiveness and scientific nature of the study. Third, more confounding factors need to be taken into account, which may be the main reason why some of our findings are contrary to some similar studies. In the future, the collection of the indicator of green space could be expanded beyond the NDVI, and consideration could be given to incorporating more green space characteristics into the study indicators, such as vegetation density, vegetation structure, plant color, and odor. In addition, other confounding factors that may affect students could be considered in future studies, such as sleep quality and substance use.

## 5 Conclusion

This study examined the pathways of action between campus GSs and college student anxiety, using campuses in Nanjing, China, as an example. Different from other studies, we developed a comprehensive theoretical framework derived from existing literature to explore the pathways between campus greenery and college student anxiety. We found that (1) campus-wide NDVI and personal 300-m range NDVI significantly affect anxiety levels in college students. (2) Campus perception, nature relatedness, and the frequency of using GS are the three pathways through which campus GSs influence students’ anxiety level; campus perception and nature relatedness are the two pathways through which individual 300-m GSs influence student anxiety. (3) Subjective perceptions are more important than objective factors in influencing mental health. We suggest that future research could focus more on the detailed characterization of green spaces and also pay more attention to the subjective perceptions of the research participants. We also hope this study will have a positive effect on the construction of campus GSs through a health-oriented approach.

## Data Availability

The raw data supporting the conclusions of this article will be made available by the authors, without undue reservation.

## Footnotes

^1^ https://api.map.baidu.com/lbsapi/getpoint/index.html
