# Supplementary material for: Estimation of mediators in the associations between campus green spaces and students’ anxiety: a case study in Nanjing
Source: Front Psychol. 2024 Oct 10;15:1396548. doi: 10.3389/fpsyg.2024.1396548 (PMC11499108; doi:10.3389/fpsyg.2024.1396548)
Supplement: Supplementary file 1 [file Table_1.DOCX]

Figure 1. Theoretical framework
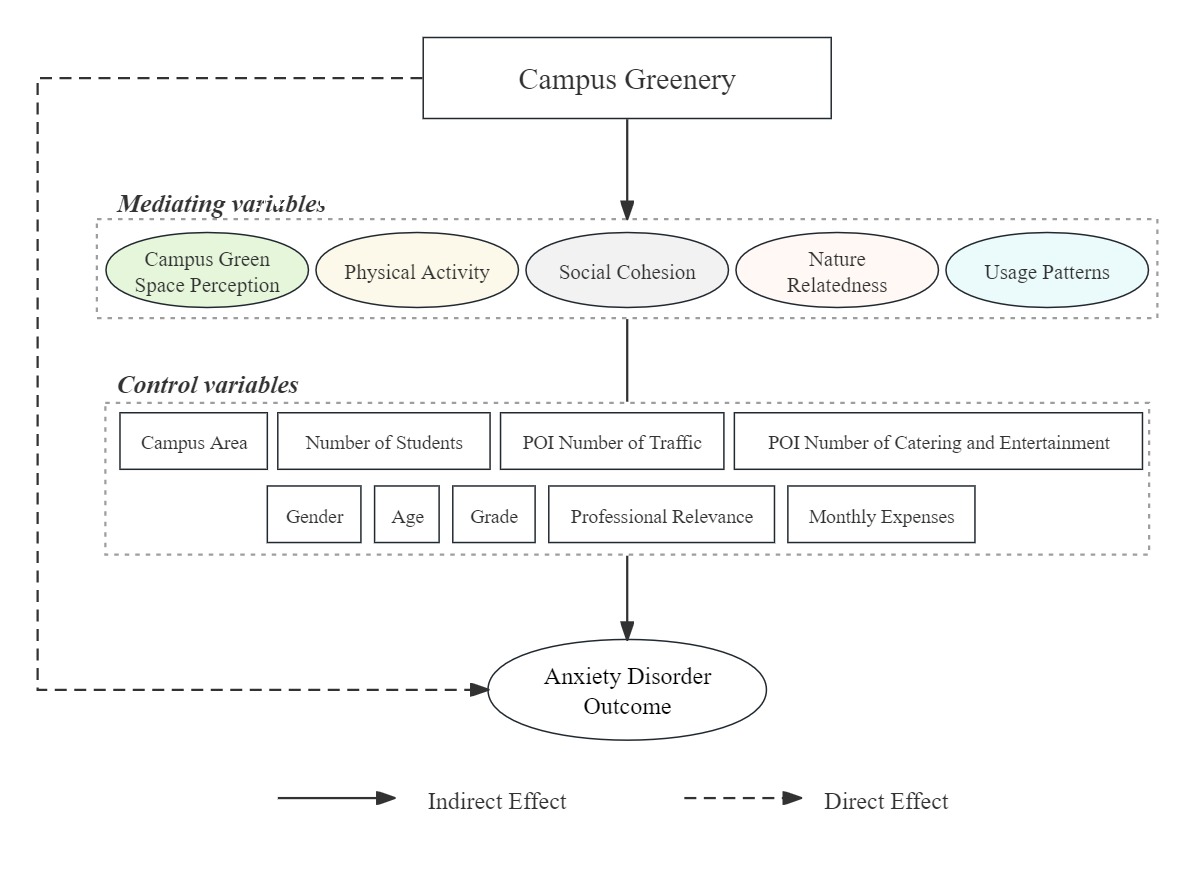


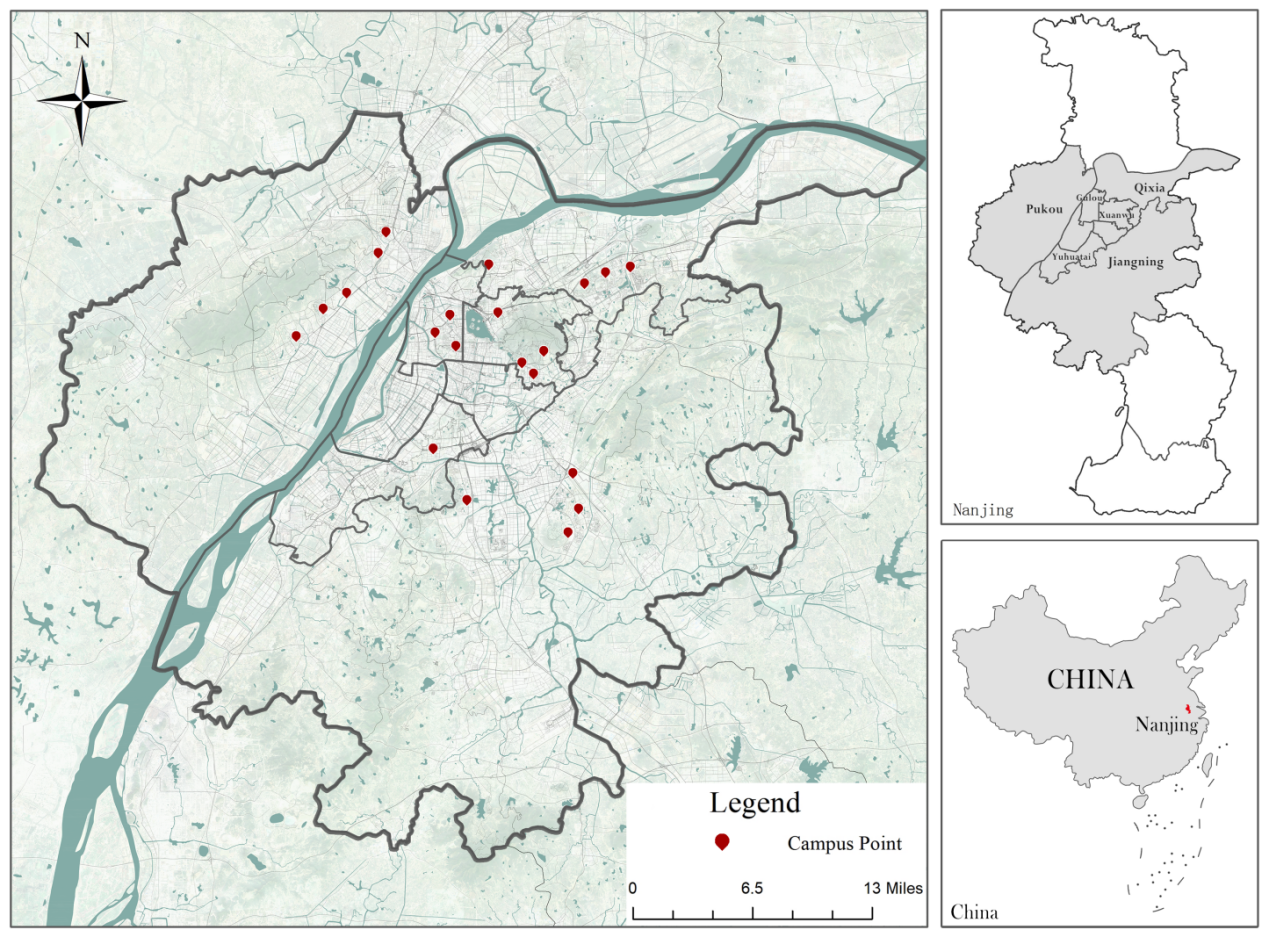


Figure 2. Locations of the 20 sampled campuses in Nanjing, China.


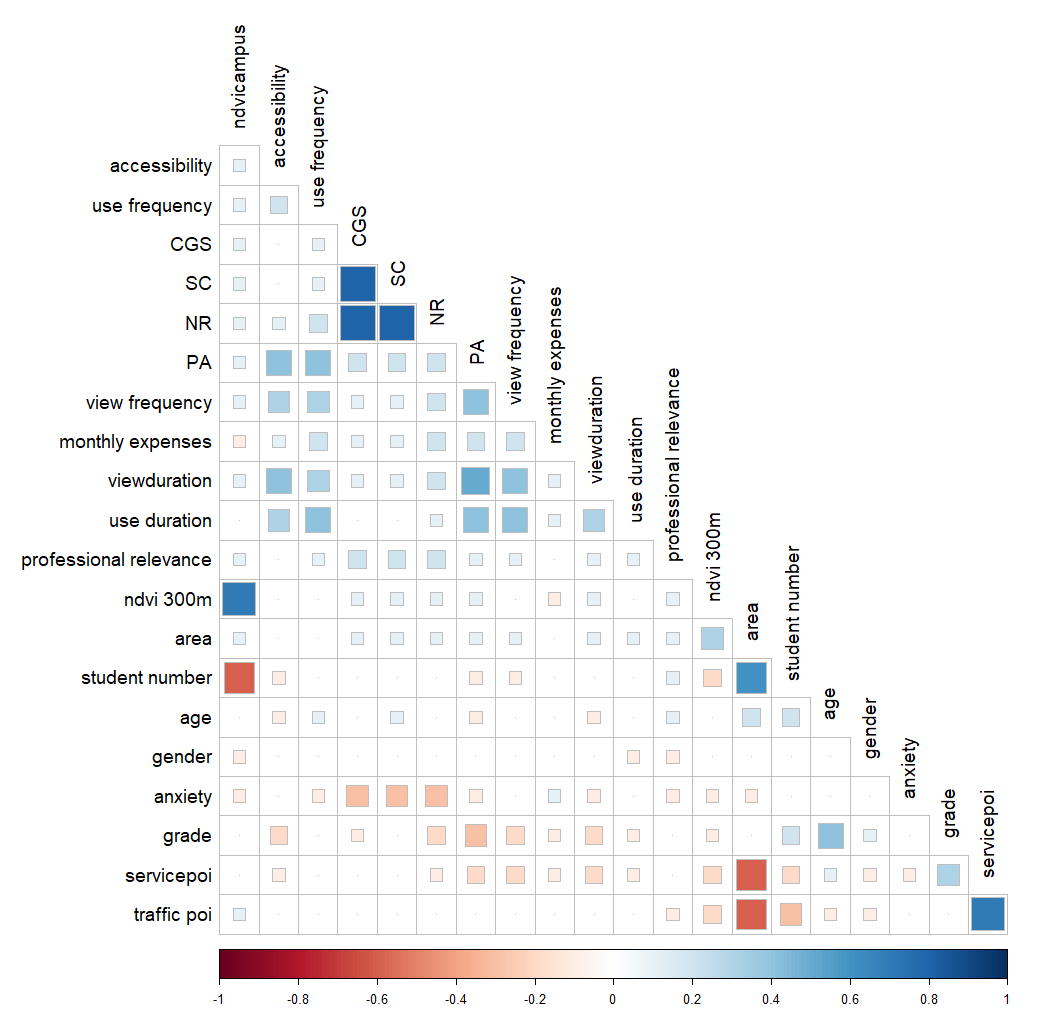


Figure 3. The Pearson correlations between variables.

Table 1. Questionnaire

**Questionnaire**

**Part 1. Respondent Sociodemographic Characteristics**

1. What’s your gender? ________ A. Male B. Female

2. What's your age? ________

3. What grade are you in? ________

5. How is the relevance of your profession to green spaces?

A. Completely irrelevant B. Relatively irrelevant C. Median D. Relatively relevant E. Completely relevant

6. What are your average monthly expense?

A. <1000 CNY B. 1000-2000 CNY C. 2000-3000 CNY

D. 3000-5000 CNY E. >5000 CNY

**Part 2. Patterns of Using Campus GS**

1. Your average frequency of your spontaneous use of campus green space in a month (active use behaviour for relaxation, socialising, exercise, etc.)
2. never B. 1-3 times a month C. once a week
3. D. 2-3 times a week E. more than three times a week
4. What is the duration of each time you use the campus green space?
5. 0 B. less than 15 minutes C. 15-30 minutes
6. D. 30-60 minutes E. more than 60 minutes
7. What is the distance you have to walk from your dorm room (or classroom, lab, library, etc., where you spend long hours each day) to reach a campus green space that you use regularly？
8. within 5 minutes B. 5-10 minutes C. 10-15 minutes
9. 15-20 minutes E. more than 20 minutes
10. On average, how many times a day do you view the greenery through your window/balcony? (Same as question 1, active use for relaxation, rest, etc.)
11. once or never B. 2-3 times C. 4-5 times D. 6-7 times E. more than 7 times
12. What is the average duration of each time you view the green space landscape from your window/balcony?
13. 1 minute or less B. 1-3 minutes C. 3-5 minutes
14. 5-10 minutes E. more than 10 minutes

**Part 3. Perceived Status**

**（1）Subjective Perception of Campus Green Space**

|  | completely disagree | quite disagree | neutral | quite agree | completely agree |
| --- | --- | --- | --- | --- | --- |
| 1. CGS1 The green space in my campus is large and comfortable | 1 | 2 | 3 | 4 | 5 |
| 2. CGS2 The green space layout of my campus is reasonable | 1 | 2 | 3 | 4 | 5 |
| 3. CGS3 The green space of my campus is beautiful. | 1 | 2 | 3 | 4 | 5 |
| 4. CGS4 There are many kinds of plants and flowers on my campus | 1 | 2 | 3 | 4 | 5 |

**（2）Physical Activities**

1. PA1 In the past seven days, how many days have you done vigorous exercise (such as lifting heavy objects, fast running, fast cycling, etc.) for more than 10 minutes?

A. 0 B.1-2 days C.3-4 days D.5-6 days E.everyday

2. PA2 In the past 7 days, on how many days did you do moderate exercise (e.g. lifting light weights, jogging, cycling slowly, playing double tennis, etc.) for 10 minutes or more?

A. 0 B.1-2 days C.3-4 days D.5-6 days E.everyday

3. PA3 In the past seven days, how many days did you walk for 10 minutes or more?

A. 0 B.1-2 days C.3-4 days D.5-6 days E.everyday

4. PA4 In the past seven days, how many days have you been sedentary for more than 6 hours?

A.everyday B.5-6 days C.3-4 days D.1-2 days E.0

Note: The International Physical Activity Questionnaire (IPAQ) - Short Form provides a comprehensive measure by categorizing physical activity into three levels: low, moderate, and high. The scoring is calculated by multiplying the MET value of an activity by the minutes spent on that activity and the number of days it is performed. The interpretation of these scores helps to identify whether an individual leads a sedentary lifestyle (low), meets basic health guidelines (moderate), or engages in a high level of physical activity that offers additional health benefits (high).

**（3）Social Cohesion**

|  | completely disagree | quite disagree | slightly disagree | neutral | slightly agree | quite agree | completely disagree |
| --- | --- | --- | --- | --- | --- | --- | --- |
| 1.SC1 Most of the people around me can be trusted not to take advantage of me; and if i'm in trouble, they'll help me. | 1 | 2 | 3 | 4 | 5 | 6 | 7 |
| 2.SC2 Most of the people around me are friendly, they care about the needs of others and help each other. | 1 | 2 | 3 | 4 | 5 | 6 | 7 |
| 3.SC3 The people around me are respectful and tolerant of each other. | 1 | 2 | 3 | 4 | 5 | 6 | 7 |

Note: The Social Cohesion Scale is a multidimensional tool designed to measure the degree of social cohesion within a community. It typically involves a series of statements that respondents rate on a Likert scale, indicating their level of agreement or disagreement. The scores are then averaged, with higher scores reflecting greater perceived social cohesion. The interpretation of these scores helps to identify the strength of social bonds within a community.

**（4）Nature** **Relatedness**

|  | completely disagree | quite disagree | neutral | quite agree | completely agree |
| --- | --- | --- | --- | --- | --- |
| 1.NR1 My ideal vacation destination would be a remote area that preserves the appearance of the natural environment. | 1 | 2 | 3 | 4 | 5 |
| 2.NR2 I always think about how my actions affect the environment. | 1 | 2 | 3 | 4 | 5 |
| 3.NR3 My connection to nature and the environment is part of my spirit. | 1 | 2 | 3 | 4 | 5 |
| 4.NR4 Wherever i am, i notice wildlife. | 1 | 2 | 3 | 4 | 5 |
| 5.NR5 My relationship with nature is an important part of who i am. | 1 | 2 | 3 | 4 | 5 |
| 6.NR6 I feel connected to all living things and the earth. | 1 | 2 | 3 | 4 | 5 |

The Nature Relatedness Scale measures an individual’s connection to nature rated on a five-point Likert scale, ranging from 1 (disagree strongly) to 5 (agree strongly). The overall score is calculated by averaging all items, with certain items reverse-scored to ensure accuracy. Higher scores indicate a stronger connection to nature.

**Part 4. Self-rated Anxiety Degree**

| In the last two weeks, how often have you experienced the following symptoms in your daily life? | 1 | 2 | 3 | 4 |
| --- | --- | --- | --- | --- |
| 1.GAD1 Feeling nervous, anxious, or eager | No | Several days | More than half the time | Almost every day |
| 2.GAD2 Can't stop or control worrying | No | Several days | More than half the time | Almost every day |
| 3.GAD3 Worrying too much about all kinds of things | No | Several days | More than half the time | Almost every day |
| 4.GAD4 Feeling hard to relax | No | Several days | More than half the time | Almost every day |
| 5.GAD5 Unable to sit still due to restlessness | No | Several days | More than half the time | Almost every day |
| 6.GAD6 Become easily annoyed or irritable | No | Several days | More than half the time | Almost every day |
| 7.GAD7 Feeling afraid that something terrible is going to happen | No | Several days | More than half the time | Almost every day |

Thank you for participating in the survey, wish you a happy life!

Table 2. Comparison of impact size of subjective and objective indicators

| **Variables** | **Model 1**  **PA** |  | **Model 2**  **SC** |  | **Model 3**  **NR** |  | **Model 4**  **GAD** |  |
| --- | --- | --- | --- | --- | --- | --- | --- | --- |
|  | **B (95%CI)** | VIF | **B (95%CI)** | VIF | **B (95%CI)** | VIF | **B (95%CI)** | VIF |
| **intercept** | 2.235^**^  (1.602,2.868) |  | 1.168^**^  (0.616,1.720) |  | 0.993^**^  (0.603,1.383) |  | 17.249^**^  (13.052,21.447) |  |
| Monthly expenses | 0.148^**^  (0.076,0.219) | 1.055 | 0.054  (-0.009,0.116) | 1.055 | 0.067^*^  (0.025,0.109) | 1.055 | 0.716^*^  (0.240,1.191) | 1.055 |
| Campus area | 0.000  (-0.001,0.000) | 1.755 | 0.001  (-0.001,0.001) | 1.755 | 0.000  (-0.001,0.000) | 1.755 | -0.008^*^  (-0.13,-0.002) | 1.755 |
| POI number of catering and entertainment | 0.000  (-0.001,0.000) | 1.680 | 0.001  (0.000,0.000) | 1.680 | 0.000  (0.000,0.000) | 1.680 | -0.03^**^  (-0.04,-0.01) | 1.680 |
| Age | -0.016  (-0.036,0.005) | 1.087 | -0.011  (-0.007,0.029) | 1.087 | -0.002  (-0.014,0.010) | 1.087 | 0.026  (-0.110,0.162) | 1.087 |
| CGS | 0.160^**^  (0.079,0.242) | 1.063 | 1.054^**^  (0.982,1.124) | 1.124 | 0.719^**^  (0.692,0.787) | 1.124 | -2.246^**^  (-2.787,-1.706) | 1.063 |
| 300m NDVI | 0.849  (-0.474,2.171) | 1.130 | -0.098  (-1.360,0.946) | 1.111 | -0.105  (-0.792,0.751) | 1.111 | -5.892  (-14.660,2.876) | 1.130 |
| R^2^ | 0.095 |  | 0.598 |  | 0.619 |  | 0.138 |  |
| Adjusted R^2^ | 0.086 |  | 0.592 |  | 0.613 |  | 0.030 |  |

Note: p<0.05*, p<0.01**
